# Building AI capability from digital orientation: the roles of organizational learning, organizational forgetting, and trust in AI

**DOI:** 10.3389/fpsyg.2026.1886459

**Published:** 2026-07-17

**Authors:** Wei Yang, Yizhuo Ma, Qiurong Yang

**Affiliations:** School of Economics and Management, Xichang University, Xichang, China

**Keywords:** AI capability, AI trust, digital orientation, organizational forgetting, organizational learning

## Abstract

Despite the growing importance of artificial intelligence in business contexts, empirical research on how firms transform digital orientation into AI capability remains limited, particularly when many firms invest in digital and AI initiatives but fail to develop a coherent capability base. Based on the attention based view, this study proposes a framework with mediation and moderation to explain the direct and indirect effects of digital orientation on AI capability, mediated by organizational learning and organizational forgetting, and moderated by the level of AI trust within the firm. We carried out a quantitative study using survey data collected in 2025 from 306 Chinese firms across different industries, ownership types and firm sizes. We applied structural equation modelling, bootstrap procedures and fuzzy set qualitative comparative analysis to examine the variable relationships and configurational paths that explain AI capability formation. The results show that digital orientation is positively associated with AI capability through organizational learning and organizational forgetting. Furthermore, we provide evidence that AI trust strengthens the association between digital orientation and these two knowledge processes, which is consistent with a stronger conversion of digital strategic attention into AI capability. The configurational analysis further reveals two routes to high AI capability, namely a trust based forgetting route and a mature firm learning route. These findings clarify the knowledge mechanism through which digital orientation becomes AI capability and highlight the joint importance of learning, forgetting and trust.

## 1 Introduction

Enterprise AI adoption has become near-universal, but advantage has not. McKinsey and Company’s (2025) State of AI survey shows that 88% of organizations now use AI in at least one business function, up from 55% in 2023. But only about 6% are high performers in measurable EBIT impact (McKinsey, 2025). The real issue is not who is using AI today, but why only a few businesses can benefit in the end from it. This paper addresses this problem using AI capabilities. Following Mikalef and Gupta (2021), AI capability means a firm’s ability to use data, technology, human skills, and organizational resources to develop and manage AI applications. Some firms can put AI into daily work, decision-making, and innovation activities. But many firms still collect digital tools without building a strong and complete capability system. In January 2025, the release of DeepSeek’s DeepSeek-R1 made this difference more clear among Chinese firms. The lower cost and lower technical requirements for AI testing changed the key management question. Firms no longer ask whether AI can be accessed. They now ask whether the organization can use AI effectively.

Two literatures bear on this question, yet they have largely developed in parallel. The first examines the antecedents of AI capability and points to data assets, technological infrastructure, human and managerial skills, dynamic capabilities and learning routines as critical conditions for AI-based value creation (Mikalef and Gupta, 2021; Ritala et al., 2024; Simón et al., 2024). The second concerns digital orientation, which captures a firm’s strategic commitment to digital technologies and has been linked to digital transformation, innovation outcomes and organizational performance (Kindermann et al., 2021; Wang et al., 2024; Wang K. et al., 2025). Each line of inquiry has produced important insights on its own terms. However, how digital orientation is actually converted into AI capability remains insufficiently specified. In particular, the internal organizational processes through which a digital strategic posture takes shape as AI-related capability have received limited theoretical and empirical attention. The unresolved puzzle, then, is not whether digital orientation matters, but why some digitally oriented firms convert that orientation into AI capability while others, equally committed on paper, do not. Recent work on AI-driven digital transformation reinforces the point: organizational AI readiness improves business-process efficiency largely through an AI-driven transformation process rather than directly (Salam et al., 2026a), which locates the decisive action in the intervening organizational processes rather than in stated commitment alone.

The puzzle just described maps onto a broader theoretical question about how firms allocate attention. The attention-based view of the firm provides a way to address it. Ocasio (1997) argues that firm behavior depends less on the full set of opportunities and threats facing the organization than on the issues decision-makers actually attend to, with attention itself shaped by how the firm structures its communication channels, rules and incentives. Strategic orientations operate within this logic. They function as filters, determining which signals enter the managerial agenda and which routines receive resources and revision, rather than serving as mere declarations of intent. Digital orientation, on this account, is a firm-level pattern of attention that pushes digital and AI-related issues into the foreground of managerial cognition. The harder question is what happens next. A pattern of attention does not automatically become competence. For digital orientation to translate into AI capability, attention must be carried into the organizational processes through which firms absorb new knowledge and revise prior assumptions. The remainder of this paper examines two such processes.

What does it take, then, for digital orientation to translate into actual AI capability? Our model identifies two parallel pathways. One runs through organizational learning. Following Sinkula et al. (1997) and Alegre and Chiva (2008), we treat learning as a firm’s capacity to experiment, acquire external knowledge, communicate openly across functions, and engage employees in solving novel problems. Digital orientation can stimulate this capacity in two ways: by elevating digital and AI-related concerns on managerial agendas, and by signaling that exploration is sanctioned at the top. The importance of this issue becomes clearer when we look at what AI use really needs. AI applications usually do not create value when they are first used. They need repeated testing, explanation of unexpected results, and improvement of data and prompts. If learning routines are weak, these steps do not happen, and the technology stays unused or only partly used. The finding from McKinsey and Company (2025) that nearly two-thirds of organizations are still in the pilot stage matches this situation. Technology has spread faster than the learning routines needed to use it well.

The other path is more debated in existing studies. Strategy researchers have argued for a long time that firms need to remove old routines that no longer fit current needs (Qu et al., 2022). But studies on AI capability have paid less attention to what firms need to remove and have focused more on what firms should build. We believe this difference is important. AI capability is usually not just an added resource. For example, data-based recommendations do not simply add new information beside experience-based judgment. They can make human judgment look biased and force firms to decide which opinion should be followed when the two sides disagree. Similar problems appear when AI workflows shorten decision time but old approval systems were designed to slow decisions down. If firms do not solve these problems by removing old routines on purpose, employees may resist the new technology, or firms may use AI only as an extra layer beside existing work practices. Digital orientation is important because it helps firms notice that some old routines should be removed. But whether firms really remove them depends on their ability to forget old practices. This ability is very different across firms, even when many firms say they strongly support digital transformation.

Both paths assume one thing that is often not true in real work situations: organizational members are willing to use AI results in their decisions. Because of this, we introduce AI trust as a boundary condition. Drawing on recent research surrounding human-AI relations by Choung et al. (2023) and Hoffman et al. (2023), we define AI trust as the degree to which managers and employees believe AI systems are reliable and useful enough to use in making real work decisions. Although our focus is the firm level, this resonates with individual-level evidence on AI acceptance: in consumer settings, usefulness expectancy together with privacy concerns shapes whether people are willing to rely on AI-enabled devices (Salam et al., 2026b), underscoring that trust functions as an enabling condition for actual reliance rather than a by-product of exposure. This condition is more important than it first appears. If AI trust exists, the learning and forgetting paths will work properly. Employees pay attention to algorithm feedback instead of ignoring it and replacing familiar routines with AI-based alternatives becomes psychologically acceptable. But if AI trust is low, the same digital orientation may only contingently produce surface compliance. Employees may review AI results but not truly depend on them. Existing routines may continue to be a backup system and formal support for digital transformation may not affect daily work practices. We therefore expect AI trust to strengthen both paths through which digital orientation affects AI capability. The effects should be weaker in low-trust environments, even when firms clearly support digital transformation.

The empirical analysis is based on survey data from 306 middle and senior managers in Chinese enterprises. Structural equation modelling is used to test the direct and mediating relationships among digital orientation, organizational learning, organizational forgetting and AI capability. Hierarchical regression and bootstrap procedures are used to examine the moderating role of AI trust and the corresponding conditional indirect effects. To complement the net effect analysis, fuzzy set qualitative comparative analysis is also conducted to identify configurations of conditions sufficient for high AI capability. The results show that digital orientation has a positive effect on AI capability. Organizational learning and organizational forgetting both mediate this relationship. AI trust strengthens the effects of digital orientation on both mediators and enhances the two indirect paths. The configurational analysis further reveals multiple routes to high AI capability, including a trust and forgetting synergy configuration and a mature firm learning driven configuration.

**Figure 1:**
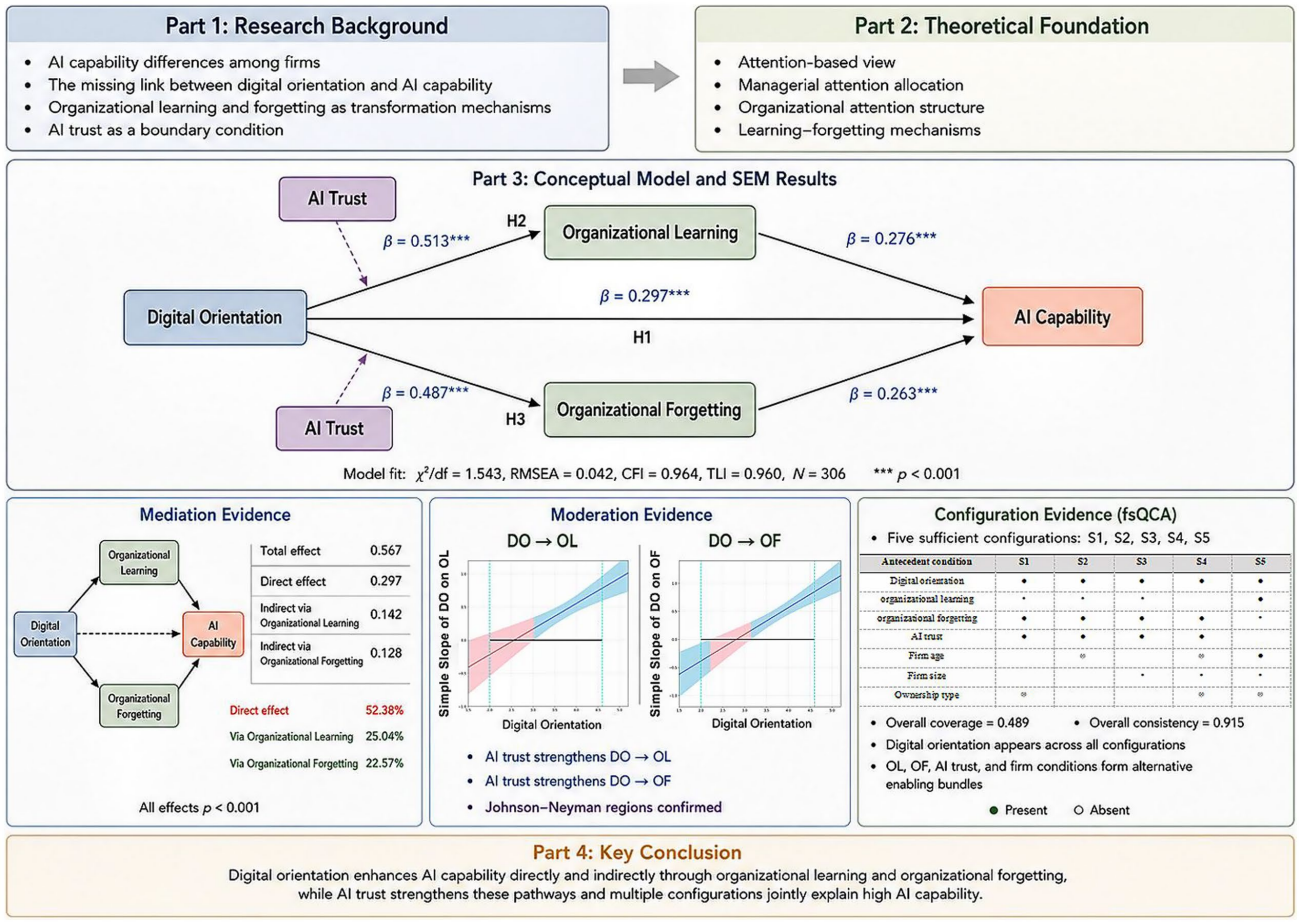
Overview of the research framework and empirical logic.

This article contributes to the literature in three ways. First, it extends research on digital orientation by showing how a digital strategic posture becomes an AI capability. Rather than treating digital orientation as a general predictor of digital transformation, the study conceptualizes it as an attentional configuration that gains organizational force through learning and forgetting processes. Second, it brings organizational learning and organizational forgetting into a common explanatory framework. AI capability is shown to depend not only on the acquisition of new digital knowledge but also on the deliberate release of obsolete routines and assumptions. Third, it advances AI capability research by identifying AI trust as a condition that governs whether digital orientation can activate the knowledge processes required for capability formation. By combining structural equation modelling with configurational analysis, the study also shows that AI capability does not follow a single linear route, but emerges from different combinations of strategy, knowledge transformation, trust and organizational context. Figure 1 summarizes the logic of the study, linking the research background, theoretical foundation, conceptual model, empirical evidence and main conclusion. It shows how the article explains uneven AI capability formation through digital orientation, organizational learning, organizational forgetting and AI trust. The research framework and empirical logic of this study are presented in Figure 1.

## 2 Theoretical background and hypotheses

### 2.1 Attention based view

The attention-based view (ABV) was first developed by Ocasio (1997) to account for how organizations identify and act on issues drawn from a far broader set of possibilities. In contrast to earlier rationalist accounts of strategic behavior, Ocasio argues that the actions an organization actually takes at any moment reflect the limited subset of issues that decision makers are attending to, rather than the complete landscape of opportunities and threats facing the firm. Because cognitive capacity is limited, attention becomes a scarce organizational resource. The way organizations allocate attention affects which strategic priorities are pursued and which are ignored (Cyert and March, 1963; Ocasio, 1997). Strategic management studies have continued to use this framework (Ocasio, 2011; Joseph and Wilson, 2018). We use this framework to study how firms develop AI capability.

Ocasio (2011) later divided this framework into three principles. The first principle is focus of attention. It refers to the limited group of issues that decision-makers actively pay attention to at a certain time. Because attention capacity is limited, this focus affects which issues enter strategic decision-making. But attention does not operate alone. The second principle is situated attention. It means that the issues people attend to are shaped by the procedures and social contexts surrounding those issues. The third principle moves the analysis from individuals to firms. Structural distribution of attention are the organizational rules, resource allocations and authority structures that shape which issues become important across departments and over time. Together, these principles describe attention as a structural feature of the firm instead of only an individual cognitive factor. This account helps to explain why firms sharing the same external change can respond at very different speeds (Ocasio and Joseph, 2005; Helfat and Peteraf, 2015).

Recent studies on digital and AI transformation have used this framework more often. Pinski et al. (2024) used upper-echelons theory and found that AI literacy among top managers affects both AI orientation and AI implementation. This happens partly because AI literacy directs managers’ attention to AI-related issues. Earlier studies show similar ideas. Eggers and Kaplan (2009) find that managerial cognition affects how firms respond to technological change. Helfat and Peteraf (2015) later extended the idea to the microfoundations of dynamic capability. For this study, digital orientation should not be seen only as a stated preference for digital projects. It should also be viewed as a firm-level attention pattern affecting how strategic goals become operational capability (Kindermann et al., 2021; Wang et al., 2024).

Framing digital orientation in this way also clarifies why the attention-based view, rather than a purely resource-based account, is the appropriate lens for our question. A resource-based view explains differences in the stock of digital and AI resources, but it is less able to explain why firms with comparable resources diverge so sharply in the AI capability they ultimately build. The attention-based view locates that divergence in how firms selectively allocate scarce managerial attention, and it supplies the cognitive microfoundation that dynamic-capability accounts tend to presuppose (Helfat and Peteraf, 2015; Ocasio et al., 2018). On this view, attention is not a static filter but is enacted through communication and organizational routines (Ocasio et al., 2018), which is precisely why a digital orientation becomes consequential only when it is carried into the knowledge processes examined below.

These features of the attention-based view inform the model in this study. First of all, they help us to grasp the idea of digital orientation. In this study, digital orientation is defined as a firm-level strategic orientation that operates as a structural pattern of attention, channeling digital and AI-related issues into the foreground of managerial cognition. So defined, it is distinct from general technology adoption and from a narrower AI orientation, and it is this attentional configuration, rather than the mere presence of digital tools, that is expected to give rise to AI capability. The same features inform our treatment of process. Attentional intent does not convert into capability automatically; it requires intermediate Organizational processes, in particular the dual work of acquiring new knowledge and discarding obsolete routines (Ritala et al., 2024; Klammer and Gueldenberg, 2020). Finally, the principle of situated attention helps explain why some attentional configurations remain dormant. Whether a digital orientation is actually enacted depends on the cognitive and affective conditions surrounding decision-makers, with trust in AI systems being a particularly relevant condition in our setting (Choung et al., 2023; Vanneste and Puranam, 2025). These considerations jointly motivate the moderated parallel-mediation model developed in the next section.

### 2.2 Hypotheses

#### 2.2.1 Digital orientation and AI capability

Capability formation, on the attention-based view, begins with attention. Whether a firm sustains managerial focus on digital and AI-related issues largely determines whether the underlying competences ever come together. Mikalef and Gupta (2021) describe AI capability as a bundle of resources spanning technical inputs such as data preparation, infrastructure and AI-specific skills, alongside organizational ones such as managerial understanding of business problems, recognition of new opportunities and support for organizational change. Resources of this kind do not emerge from fragmented or episodic technology initiatives. They presuppose a strategic orientation that keeps digital transformation continuously on the managerial agenda and channels resources toward it.

Digital orientation supplies this strategic foundation. Following Kindermann et al. (2021) and Wang et al. (2024), we treat it as a firm’s willingness to adopt and integrate digital technologies for the purpose of identifying and exploiting new opportunities. The principal characteristic is breadth. A digitally oriented firm does not treat AI as an isolated tool but as one element in a broader digital agenda that connects data infrastructure, digital talent and cross-functional coordination to specific business problems. This is what allows digital orientation to convert technological awareness into capability rather than letting the two remain disconnected.

Recent studies have also shown that digital orientation is associated with a range of capability building outcomes. Wang et al. (2024) find that digital orientation enhances exploratory innovation through external knowledge acquisition. Wang K. et al. (2025) show that digital orientation improves firm outcomes through coordination structures. Pinski et al. (2024) further demonstrate that AI literacy at the top management level strengthens both AI orientation and AI implementation. Taken together, these studies suggest that digital orientation configures firm attention in ways that support the accumulation of AI capability. Accordingly, we propose:

*H1*: Digital orientation is positively associated with AI capability.

#### 2.2.2 The mediating role of organizational learning

Digital orientation is likely to foster AI capability through organizational learning. Organizational learning refers to a firm’s capacity to experiment with new approaches, tolerate calculated risk, absorb external knowledge, sustain open communication and involve members in decision making (Alegre and Chiva, 2008; Sinkula et al., 1997). These learning activities are particularly important in digital transformation because AI related knowledge is often complex, fast changing and distributed across technological and business domains.

A firm with a strong digital orientation is more likely to activate these learning routines. When digital and AI issues become regular items on the managerial agenda, exploration of new digital tools is treated as a normal part of employee work. It is not treated as optional experimentation. Knowledge also moves more easily across departments. External partners move from occasional contacts to direct sources of technological insight. Konopik et al. (2022) make a similar point. They say successful digital transformation depends on a firm’s ability to learn, coordinate, and adapt. Ivaldi et al. (2022) make the same point from a workplace culture perspective. They show that new digital work cultures depend on learning processes. These processes affected individual skills and group routines.

Organizational learning converts these conditions into AI capability. AI capability usually does not come directly from buying AI tools or setting up digital systems. It needs repeated testing and reflection. Firms learn how to prepare data, how to interpret model results, how to re-architect workflows to incorporate AI, and how to tie incorporation of AI to real business problems. Recent studies support this view. Ritala et al. (2024) argue that industrial AI capability originates from organizational learning, not just from technology investment. Simón et al. (2024) find that AI value creation is contingent on human-AI teamwork. Braojos et al. (2024) found digital leadership affects AI-related capability through continuous learning environment. Organizational learning accounts for much of this effect. We propose:

*H2*: Organizational learning mediates the positive relationship between digital orientation and AI capability.

#### 2.2.3 The mediating role of organizational forgetting

Learning alone is usually not enough, because acquiring new knowledge and discarding obsolete knowledge are distinct operations. Organizational learning is fundamentally additive, involving the acquisition, creation, and retention of new routines, whereas organizational forgetting is subtractive, referring to the intentional and often costly relinquishment of incumbent routines that have become liabilities rather than assets (Tsang and Zahra, 2008). Forgetting therefore cannot be reduced to the absence of learning. Learning can proceed by layering new practices onto established ones, but forgetting acts on those established practices themselves and obliges members to give up prior competence, so that the two processes vary independently and occupy separate dimensions of intentionality and depth of knowledge loss (Klammer and Gueldenberg, 2019). It is this subtractive work that AI most often demands. Following Qu et al. (2022), organizational forgetting means purposeful work to remove routines, processes, and knowledge structures when they become barriers to renewal, not useful assets. In adopting AI, firms often need to be willing to give up old routines based on managerial intuition and scattered data. They also need to give up manual procedures and decision-making that stays within separate functions. Without deliberate forgetting, AI tools are merely layered on top of old routines. The firm gains technology but it does not build real capability.

A digital orientation makes this process more likely. When digital transformation becomes a priority, the organization starts to change what it considers useful or acceptable. Routines that had provided stability start to be viewed as limits. Klammer and Gueldenberg (2020) describe this two-part process. They say innovation requires firms to retain useful old knowledge and drop practices that hinder renewal. Grisold et al. (2020) explain this by demonstrating different forms of organizational unlearning. These forms often appear when firms face new strategic needs.

AI makes organizational forgetting more important than many of its earlier technologies because AI applications often replace old practices instead of working side by side with them. Data-driven recommendation systems may challenge experience-based judgment. AI enabled workflows may weaken traditional functional boundaries. Predictive analytics may replace rule based forecasting. These changes require firms to abandon certain assumptions and routines before AI capability can become embedded in organizational practice. Kluge (2023) concludes that intentional forgetting and unlearning can support innovation and renewal across individual, team and organizational levels. Shahzad et al. (2024) similarly find that organizational forgetting facilitates innovation by strengthening absorptive processes. Therefore, organizational forgetting should function as another mechanism through which digital orientation supports AI capability. Accordingly, we propose:

*H3*: Organizational forgetting mediates the positive relationship between digital orientation and AI capability.

#### 2.2.4 The moderating role of AI trust in the learning path

The effect of digital orientation on organizational learning is unlikely to be uniform across firms. From an attention based view, strategic attention must be enacted in specific cognitive and social contexts before it can shape organizational behavior (Ocasio, 2011). In AI related transformation, one of the most important contextual conditions is AI trust. AI trust refers to organizational members’ belief that AI systems are reliable, predictable, effective and credible enough to be incorporated into work decisions (Choung et al., 2023; Hoffman et al., 2023). Trust of this kind has both a cognitive component, grounded in perceptions of reliability, transparency, and competence, and an emotional component shaped by experience and affect (Glikson and Woolley, 2020). We model AI trust as a first-stage contextual moderator rather than as an antecedent or a mediator for a specific theoretical reason: trust does not itself generate a firm’s digital orientation, but, consistent with the principle of situated attention, it conditions whether that orientation is enacted into the knowledge processes through which capability forms. In this sense, trust sets the gain on the two pathways rather than initiating them.

AI trust is likely to strengthen the relationship between digital orientation and organizational learning. When employees and managers trust AI systems, they are more willing to test AI recommendations, incorporate AI outputs into discussions, learn from algorithmic feedback and refine work practices through human AI interaction. In contrast, when trust in AI is low, employees may treat AI outputs with suspicion, avoid experimentation or use AI only superficially. Qualitative evidence sharpens this contrast: distinct configurations of cognitive and emotional trust lead members either to rely on AI actively or to manipulate, confine, or withdraw their inputs, with corresponding consequences for how much the organization learns from the technology (Vuori et al., 2024). Gillespie et al. (2025) show that trust shapes the acceptance of artificial intelligence, while Arias-Pérez and Vélez-Jaramillo (2022) find that anxiety about AI can weaken the positive role of digital orientation in digital innovation. Thus, AI trust should help firms translate digital orientation into stronger organizational learning. Accordingly, we propose:

*H4*: AI trust positively moderates the relationship between digital orientation and organizational learning, such that the relationship is stronger when AI trust is higher.

#### 2.2.5 The moderating role of AI trust in the forgetting path

AI trust should also condition the effect of digital orientation on organizational forgetting. Forgetting established routines is often more difficult than acquiring new knowledge because it requires members to relinquish familiar practices, question prior competence and accept uncertainty about alternative ways of working (Klammer and Gueldenberg, 2020; Grisold et al., 2020). In the AI context, employees and managers are unlikely to abandon established routines unless they believe that AI mediated practices are sufficiently reliable and useful.

Whether digital orientation actually translates into organizational forgetting depends on whether members trust AI to fill the space left behind. In high-trust environments, AI outputs and AI-enabled processes are treated as credible substitutes for the routines they replace, which lowers the psychological cost of giving up established practices and makes the conversion of digital orientation into actual forgetting more likely. In low-trust situations the opposite is typically true. We describe formal compliance with AI tools coexisting with informal retention of legacy routines: employees adopt the technology on paper while keeping the old ways available as a fallback. The visible appearance of digital transformation is preserved, but the underlying routines remain intact. Vanneste and Puranam (2025) trace this logic to the individual level, arguing that trust in artificial intelligence shapes both perceived agency and the willingness to rely on AI outputs. Habbal et al. (2024) build on the point to the organizational level, finding that trust separates firms that commit to deeper AI transformation from those that accept reversible or symbolic adaptation. Taken together, these arguments suggest that AI trust strengthens the path from digital orientation through organizational forgetting to AI capability. We therefore propose:

*H5*: AI trust positively moderates the relationship between digital orientation and organizational forgetting, such that the relationship is stronger when AI trust is higher.

#### 2.2.6 The moderated mediation effects of AI trust

AI trust does more than affect the two first-stage relationships one by one. It also shapes the full path from digital orientation to AI capability. Digital orientation may improve AI capability through organizational learning and organizational forgetting. AI trust affects how strongly each path works. So, the indirect effect of digital orientation on AI capability may change when the level of AI trust in the firm changes.

First, consider the learning path. In high-trust firms, employees are more open to trying AI. They are also more willing to use AI outputs in their work. They are more likely to see what they learn from AI as real organizational knowledge, not as uncertain or questionable information. This leads to stronger organizational learning, and this learning helps firms build stronger AI capability. In low-trust firms, this process becomes weaker at each step.

The forgetting path works in a similar way, but the reason is different. Trust matters because employees will only give up old routines when they believe AI-based alternatives are reliable enough to replace them. When trust is high, employees are more willing to accept these alternatives, so organizational forgetting becomes faster. When trust is low, old routines stay in place because they feel safer than the new AI-based options.

Several related studies support this idea, but they do not fully explain it. Mollah et al. (2023) show a path from digital leadership to sustainable performance through organizational learning and IT capability. But their model treats this path as the same across firms, and it does not explain the emotional conditions that affect whether the path works. Tursunbayeva and Chalutz-Ben Gal (2024) study AI adoption through their TOP framework. They show that organizational trust climate is important for adoption success, but they do not include trust in a formal moderated mediation model. Bauwens and Cortellazzo (2025) also point out that the emotional and institutional context around digital leadership is still not fully studied in capability research. This study addresses this gap by treating AI trust as a first-stage moderator of two parallel indirect paths. We therefore propose:

*H6*: AI trust positively moderates the indirect effect of digital orientation on AI capability through organizational learning, such that the indirect effect is stronger when AI trust is higher.

*H7*: AI trust positively moderates the indirect effect of digital orientation on AI capability through organizational forgetting, such that the indirect effect is stronger when AI trust is higher.

**Figure 2:**
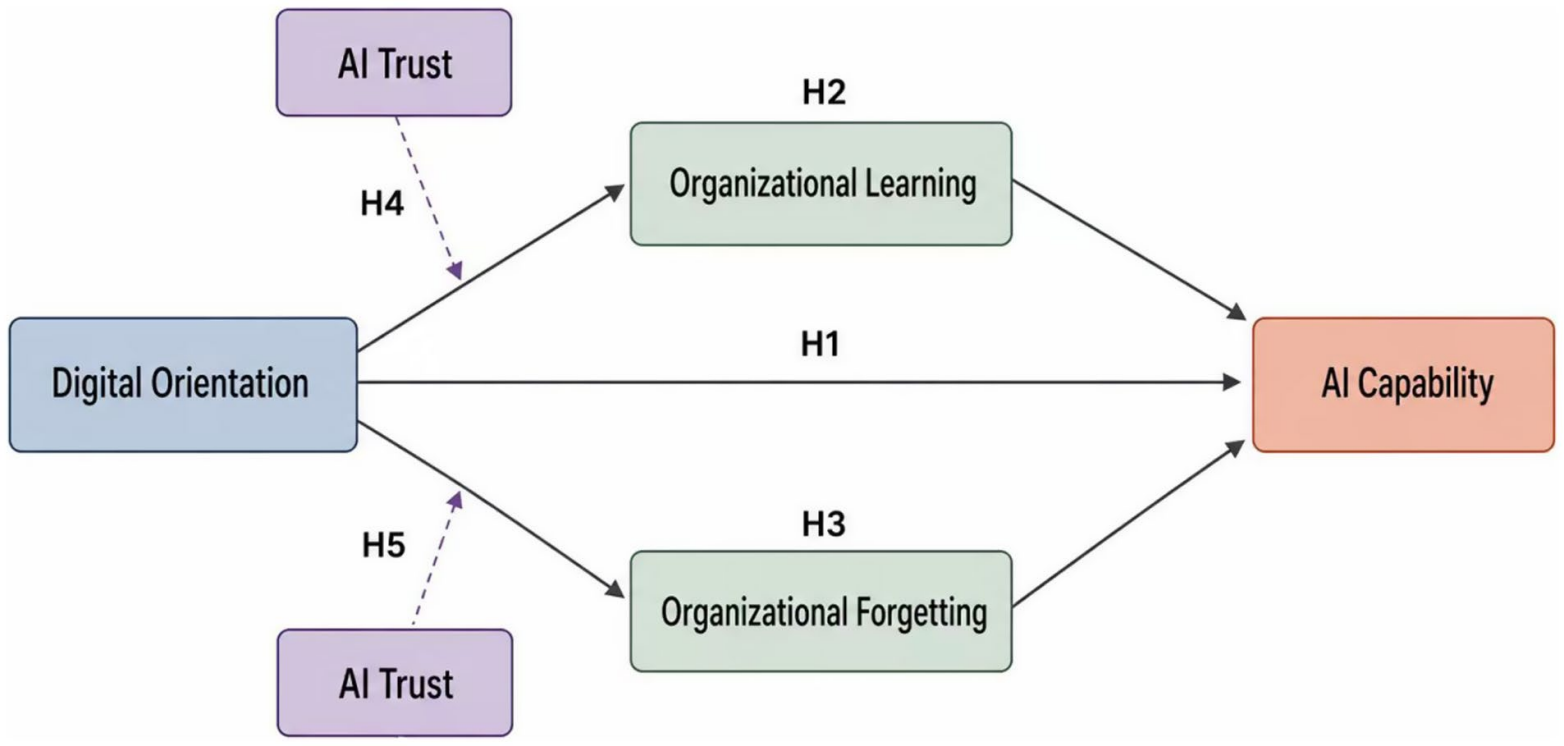
Conceptual research model. Hypotheses H6 and H7, which posit moderated mediation effects, are implied by the joint operation of H2 with H4 and of H3 with H5, and are therefore not depicted as separate paths.

Drawing on the preceding theoretical reasoning, we develop an integrated research model that links digital orientation, Organizational learning, Organizational forgetting, AI trust and AI capability. Figure 2 presents the conceptual framework.

## 3 Methodology

### 3.1 Questionnaire and variable measurement

The instruments employed in this research were derived from well-validated measurement frameworks widely used in the fields of management and information systems. To maintain linguistic equivalence between the source and target version of the questionnaire items were translated from English to Chinese and then back to English by a team of independent bilingual experts following the back-translation procedure suggested by Brislin (1970). This procedure was used to verify that the translated items maintained the content of the original items. A preliminary survey was conducted involving 25 managers to assess the clarity and appropriate context of the Chinese wording, and a number of the statements were revised to eliminate potential misunderstanding. All item responses to the measurement items were collected on a five-point Likert-type scale (1 = strongly disagree to 5 = strongly agree). Descriptions of how each of the constructs was operationalized are provided in the section below.

#### 3.1.1 Digital orientation

Following Khin and Ho (2019) and Wang et al. (2024), digital orientation was measured using a five-item scale that captures a firm’s strategic propensity to adopt, renew and integrate digital technologies in pursuit of innovation opportunities. Sample items include “We have a strong willingness to adopt and renew digital technologies” and “We use digital technologies for integrating customer information from different contact points.”

#### 3.1.2 AI capability

Following Mikalef and Gupta (2021), AI capability was measured using a six-item scale that captures a firm’s integrated competence in deploying data, technology, human skills and Organizational resources for the development and application of AI. Sample items include “Our managers are able to understand business problems and to direct AI initiatives to solve them” and “We are able to prepare and cleanse AI data efficiently and assess data for errors.” Because the scale is pitched at the level of firm-wide AI competence rather than any sector-specific application, the same six items were administered identically to all respondents, so that AI capability is captured at a comparable, firm-wide level across the mixed-industry sample.

#### 3.1.3 Organizational learning

Following Alegre and Chiva (2008), and informed by the Organizational learning framework of Sinkula et al. (1997), Organizational learning was measured using a five-item scale capturing experimentation, risk tolerance, external knowledge interaction, open communication and participative decision-making. Sample items include “Our organization encourages employees to experiment with new digital ideas and work methods” and “There is free and open communication across departments when discussing digital technologies and business problems.”

#### 3.1.4 Organizational forgetting

Following Qu et al. (2022), Organizational forgetting was measured using a five-item scale that captures a firm’s capacity to actively identify, reflect upon and discard outdated knowledge, processes and routines that no longer fit digital development. Sample items include “The firm often reviews corporate work processes and points out inadequacies” and “The firm often criticizes outdated ways of working.”

#### 3.1.5 AI trust

Following Shamim et al. (2023), and informed by Hoffman et al. (2023), AI trust was measured using a five-item scale that captures Organizational members’ confidence in the reliability, predictability, effectiveness and credibility of AI systems and their outputs. Sample items include “The AI systems used in our organization are reliable” and “I believe that the recommendations or results generated by AI systems in our organization are trustworthy.”

#### 3.1.6 Control variables

Drawing on prior research on AI capability and digital transformation (e.g., Mikalef and Gupta, 2021; Wang et al., 2024), six respondent- and firm-level characteristics that could confound the focal relationships were entered as covariates in all subsequent analyses: managerial position, age, educational attainment, firm age, ownership type and firm size.

### 3.2 Sample and data collection

Data were collected through a structured questionnaire administered to middle and senior managers in Chinese firms. The sample includes firms from different industries, ownership type and firm size. We chose managers at these levels because they can see both strategic goals and daily work practices. Examining digital orientation, learning and forgetting habits, AI capacity, and the trust environment surrounding AI adoption requires this viewpoint. The survey was shared from September to December 2025 through online channels and the authors’ professional networks. In practice, initial contacts were drawn from the authors’ academic-industry and alumni networks and from professional manager associations; each contact received a brief screening note confirming that the respondent held a middle- or senior-management role and was familiar with the firm’s digital and AI initiatives, and was invited to forward the link to qualified peers in other firms. To limit selection bias and improve coverage, we monitored the incoming distribution and actively sought additional responses so that the sample spanned a range of industries, ownership types, and firm sizes rather than clustering in a single segment. We nonetheless acknowledge that this is a non-probability, partly referral-based sample, and we return to its implications for generalizability in the Limitations section.

A total of 415 questionnaires were distributed, and 366 were returned. Responses with repeated answer patterns, too many missing values, or unrealistically short completion times were removed. The final dataset comprised 306 valid cases with an effective response rate of 73.73%. The sample is balanced between managerial levels. Senior managers make up 49.0% and middle managers 51.0%. The largest age group is 41–50 with 46.1% of respondents. More than three-quarters of the respondents hold a degree at the secondary education level or above, representing 76.1%. Privately-owned firms form the largest ownership category (69.0%), firms with fewer than 100 employees make up 55.6% of the sample, and firm age is distributed relatively evenly across the five cohorts, from 26.5% for newly established firms (≤5 years) to 15.7% for mature firms (≥21 years).

## 4 Empirical analysis

### 4.1 Reliability and validity test

The psychometric qualities of the measurement instruments were examined using a range of validity and reliability tests in a battery of hypothesis testing in Mplus 8.3. The analyses encompassed indicator reliability, internal consistency, common method variance, the factorial structure of the five focal constructs, and convergent and discriminant validity.

As reported in Table 1, Cronbach’s *α* and composite reliability (CR) values for the five focal constructs ranged from 0.869 to 0.900 and from 0.871 to 0.902 respectively, all comfortably exceeding the recommended threshold of 0.70 (Hair et al., 2019). All standardized factor loadings were significant at the *p* < 0.001 level and ranged from 0.683 to 0.852, comfortably above the 0.50 threshold for item-level convergent validity.

**Table 1:** Reliability and validity analysis.

Construct	Item	Factor loading	Cronbach’s *α*	CR	AVE
Digital orientation	DO1	0.775	0.869	0.871	0.574
	DO2	0.810			
	DO3	0.742			
	DO4	0.746			
	DO5	0.713			
AI trust	AIT1	0.776	0.900	0.902	0.647
	AIT2	0.852			
	AIT3	0.804			
	AIT4	0.809			
	AIT5	0.779			
Organizational learning	OL1	0.799	0.882	0.883	0.601
	OL2	0.757			
	OL3	0.793			
	OL4	0.779			
	OL5	0.746			
Organizational forgetting	OF1	0.744	0.895	0.895	0.631
	OF2	0.773			
	OF3	0.804			
	OF4	0.834			
	OF5	0.815			
AI capability	AIC1	0.803	0.880	0.881	0.554
	AIC2	0.683			
	AIC3	0.725			
	AIC4	0.788			
AI capability	AIC5	0.723			
	AIC6	0.738			

To assess common method variance, we applied Harman’s single-factor test by submitting all 26 measurement items to an unrotated exploratory factor analysis. The first extracted factor explained 34.07% of the total variance, well below the conventional threshold and indicating that common method bias is not a serious concern in this study (Podsakoff et al., 2003). We then verified the factorial structure of the five constructs through a sequence of competing confirmatory factor models, with the comparison reported in Table 2. The hypothesized five-factor model produced excellent fit (χ^2^/df = 1.406, RMSEA = 0.036, CFI = 0.973, TLI = 0.970) and outperformed every alternative specification in which two or more constructs were merged; the fit indices deteriorated monotonically as the level of factor consolidation increased.

**Table 2:** Confirmatory factor analysis: model comparison.

Model	Factor structure	χ^2^/df	RMSEA	CFI	TLI
Five-factor	DO, AIT, OL, OF, AIC	1.406	0.036	0.973	0.970
Four-factor	DO, AIT, OF, OL + AIC	2.888	0.079	0.875	0.861
Three-factor	DO, AIT, OF + OL + AIC	4.379	0.105	0.773	0.751
Two-factor	AIT, DO + OF + OL + AIC	5.613	0.123	0.689	0.660
One-factor	AIT + DO + OF + OL + AIC	8.193	0.153	0.513	0.470

The five-factor model is the hypothesized measurement model.

Convergent validity received further support from the average variance extracted (AVE), which fell between 0.554 and 0.647 and thus exceeded the 0.50 benchmark for every construct. Discriminant validity was confirmed through the Fornell–Larcker criterion: as Table 3 shows, the square root of each construct’s AVE on the diagonal exceeded its correlations with all other constructs. Together, these results indicate that the measurement instruments possess sound psychometric properties for the subsequent hypothesis tests.

**Table 3:** Means, standard deviations and correlations.

Variable	*M*	SD	1	2	3	4	5
1. DO	3.592	0.473	0.758				
2. OL	3.743	0.558	0.426**	0.775			
3. OF	3.297	0.547	0.406**	0.463**	0.794		
4. AIT	3.635	0.577	0.234**	0.270**	0.204**	0.804	
5. AIC	3.846	0.529	0.480**	0.487**	0.474**	0.252**	0.744

*N* = 306. **p < 0.01.

### 4.2 Descriptive statistics and correlation analysis

Table 3 reports the means, standard deviations and bivariate correlations of the focal constructs, with the square roots of the AVEs displayed on the diagonal. The means of the five constructs ranged from 3.297 for Organizational forgetting to 3.846 for AI capability on a five-point scale, indicating moderately positive levels across the focal variables, and the standard deviations all fell between 0.473 and 0.577, suggesting comparable dispersion. All bivariate correlations among the focal constructs were positive and statistically significant at the *p* < 0.01 level: digital orientation correlated most strongly with AI capability (*r* = 0.480), and AI capability also showed substantive correlations with Organizational learning (*r* = 0.487) and Organizational forgetting (*r* = 0.474). These patterns provide preliminary support for the hypothesized relationships and motivate the formal tests below.

### 4.3 Hypothesis testing

#### 4.3.1 Direct and mediating effects of digital orientation on AI capability

The hypothesized structural model was estimated in Mplus 8.3 using maximum likelihood estimation with robust standard errors, and the indirect effects were tested through the bias-corrected bootstrap procedure with 5,000 resamples (Hayes, 2018). The model achieved good fit (χ^2^/df = 1.543, RMSEA = 0.042, CFI 0.964, TLI = 0.960), satisfying the conventional thresholds (Hu and Bentler, 1999).

As reported in Table 4, digital orientation was significantly and positively associated with AI capability (*β* = 0.297, *p* < 0.001), supporting H1. The two first-stage paths from digital orientation to Organizational learning (*β* = 0.513, *p* < 0.001) and to Organizational forgetting (*β* = 0.487, *p* < 0.001) were both highly significant, as were the second-stage paths from Organizational learning (*β* = 0.276, *p* < 0.001) and Organizational forgetting (*β* = 0.263, *p* < 0.001) to AI capability.

**Table 4:** Structural model path coefficients.

Path	*β*	S.E.	C.R.	*p*
DO → OL	0.513	0.056	9.132	< 0.001
DO → OF	0.487	0.063	7.667	< 0.001
DO → AIC	0.297	0.070	4.254	<0.001
OL → AIC	0.276	0.073	3.807	<0.001
OF → AIC	0.263	0.069	3.802	<0.001

**Figure 3:**
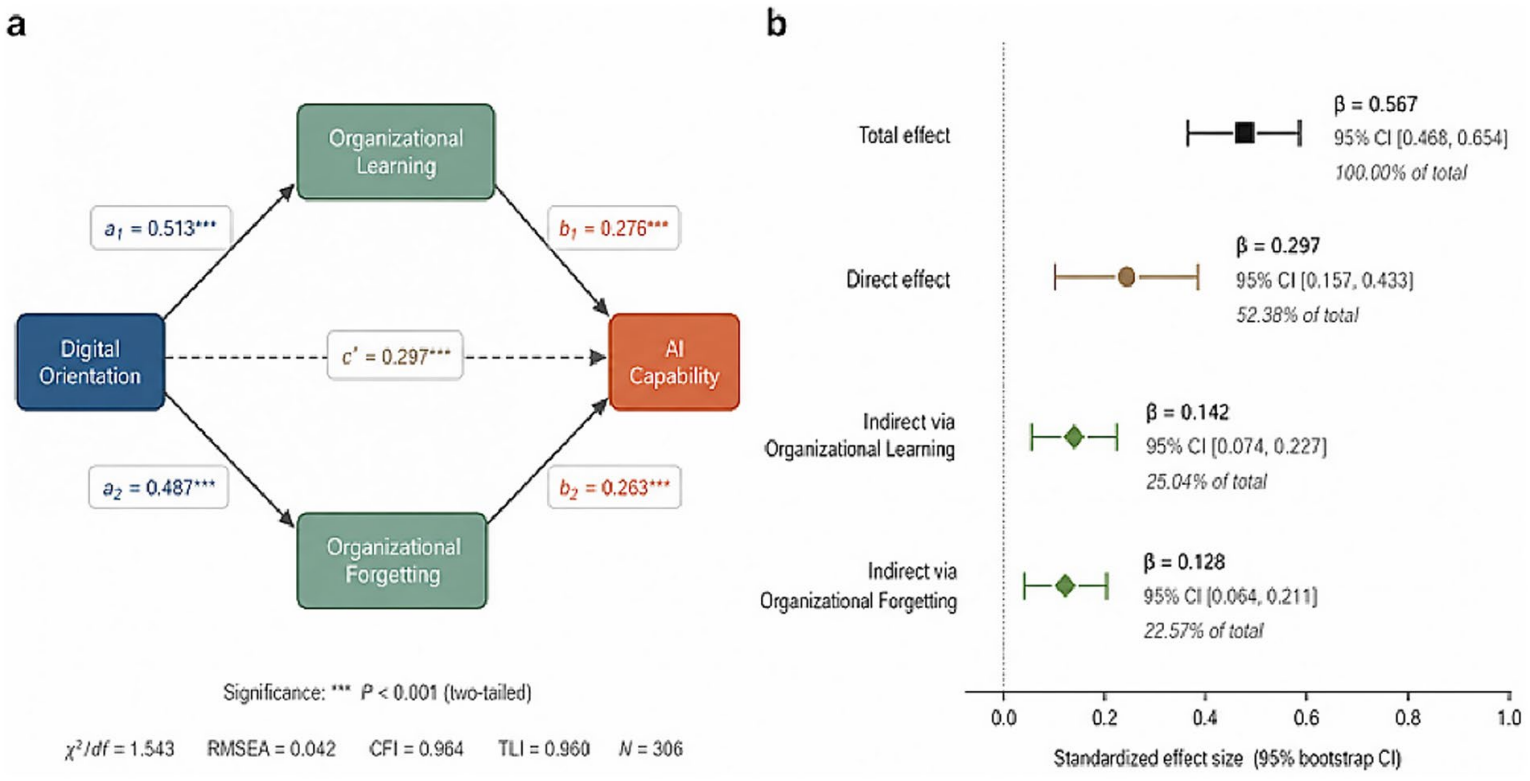
Parallel mediation analysis of digital orientation on AI capability: structural model **(a)** and bootstrap decomposition of effects **(b)**. **(a)** Reports standardized path coefficients of the structural model with model fit indices below the diagram (***p < 0.001, two-tailed). **(b)** Reports the bootstrap-based decomposition of the total effect; error bars represent 95% bias-corrected confidence intervals; percentages indicate each effect’s share of the total effect.

Bootstrap mediation results in two views (Figure 3). Figure 3a displays the parallel mediation structure as a path diagram with the first stage and second stage coefficients on each mediator close in size and the model fit indices reported below. It shows the first-stage and second-stage coefficients for each mediator. These coefficients are similar in size. The model fit indices are shown below the diagram. Figure 3b shows the bootstrap breakdown of the total effect. The total effect is *β* = 0.567. The Bootstrap 95% CI is [0.468, 0.654]. The indirect effect through Organizational learning is *β* = 0.142 (Bootstrap 95% CI = [0.074, 0.227]), accounting for 25.04% of the total. Through Organizational forgetting, the indirect effect is *β* = 0.128 (Bootstrap 95% CI = [0.064, 0.211]), or 22.57%. Both confidence intervals exclude zero, so H2 and H3 are supported. Neither mediator dominates: the two indirect paths are of nearly equal magnitude, while the direct path retains the largest share at 52.38% (see Table 5). Organizational learning and Organizational forgetting therefore operate as partial mediators of the focal relationship.

**Table 5:** Bootstrap mediation effect test.

Path	Effect	S.E.	Bootstrap 95% CI	Proportion
Indirect effects				
DO → OL → AIC	0.142	0.039	[0.074, 0.227]	25.04%
DO → OF → AIC	0.128	0.038	[0.064, 0.211]	22.57%
Direct effect				
DO → AIC	0.297	0.070	[0.157, 0.433]	52.38%
Total effect				
DO → AIC	0.567	0.047	[0.468, 0.654]	100.00%

#### 4.3.2 The moderating role of AI trust

To examine the moderating role of AI trust on the first-stage paths from digital orientation to Organizational learning and Organizational forgetting, we conducted hierarchical regression analyses in three steps: control variables were entered in Model 1, the predictor and the mean-centred moderator in Model 2, and the interaction term in Model 3. We estimate the mediation within a structural equation model so that measurement error in the latent constructs is corrected, and we estimate the moderation and the subsequent conditional effects using regression-based conditional-process analysis, which provides a transparent and readily interpretable basis for the Johnson–Neyman probing reported below. This pairing of SEM with regression-based conditional-process analysis is common in the literature, and, as shown in the following sections, the two approaches yield consistent conclusions. The results appear in Table 6.

**Table 6:** Hierarchical regression analyses for the moderating role of AI trust.

Variable	OL			OF		
	Model 1	Model 2	Model 3	Model 1	Model 2	Model 3
Constant	3.523***	1.505***	1.420***	3.416***	1.784***	1.690***
Position	0.077	0.063	0.093	0.031	0.021	0.055
Age	−0.059	−0.024	−0.005	−0.156	−0.125	−0.103
Education	0.054	−0.015	−0.018	0.069	0.011	0.007
Firm age	0.005	−0.013	−0.011	0.018	0.003	0.005
Ownership type	0.026	0.016	0.017	−0.037	−0.045	−0.045
Firm size	0.008	−0.005	−0.009	0.020	0.011	0.006
DO		0.452***	0.418***		0.386***	0.348***
AIT		0.176**	0.204***		0.122*	0.153**
DO × AIT			0.399***			0.442***
*R* ^2^	0.024	0.220	0.268	0.095	0.231	0.293
Δ*R*^2^		0.196	0.048		0.136	0.062
*F*	1.208	10.464***	12.044***	5.227***	11.144***	13.604***

*p < 0.05; **p < 0.01; ***p < 0.001.

For Organizational learning, both digital orientation and AI trust produced significant positive main effects (β_DO = 0.452, *p* < 0.001; β_AIT = 0.176, *p* < 0.01; Δ*R*^2^ = 0.196), and their interaction term yielded significant additional explanatory power (β_DO × AIT = 0.399, *p* < 0.001; Δ*R*^2^ = 0.048). A parallel pattern emerged for Organizational forgetting: the main effects were significant (β_DO = 0.386, p < 0.001; β_AIT = 0.122, *p* < 0.05; Δ*R*^2^ = 0.136), and the interaction term was significant and positive (β_DO × AIT = 0.442, *p* < 0.001; Δ*R*^2^ = 0.062). H4 and H5 are therefore supported.

**Figure 4:**
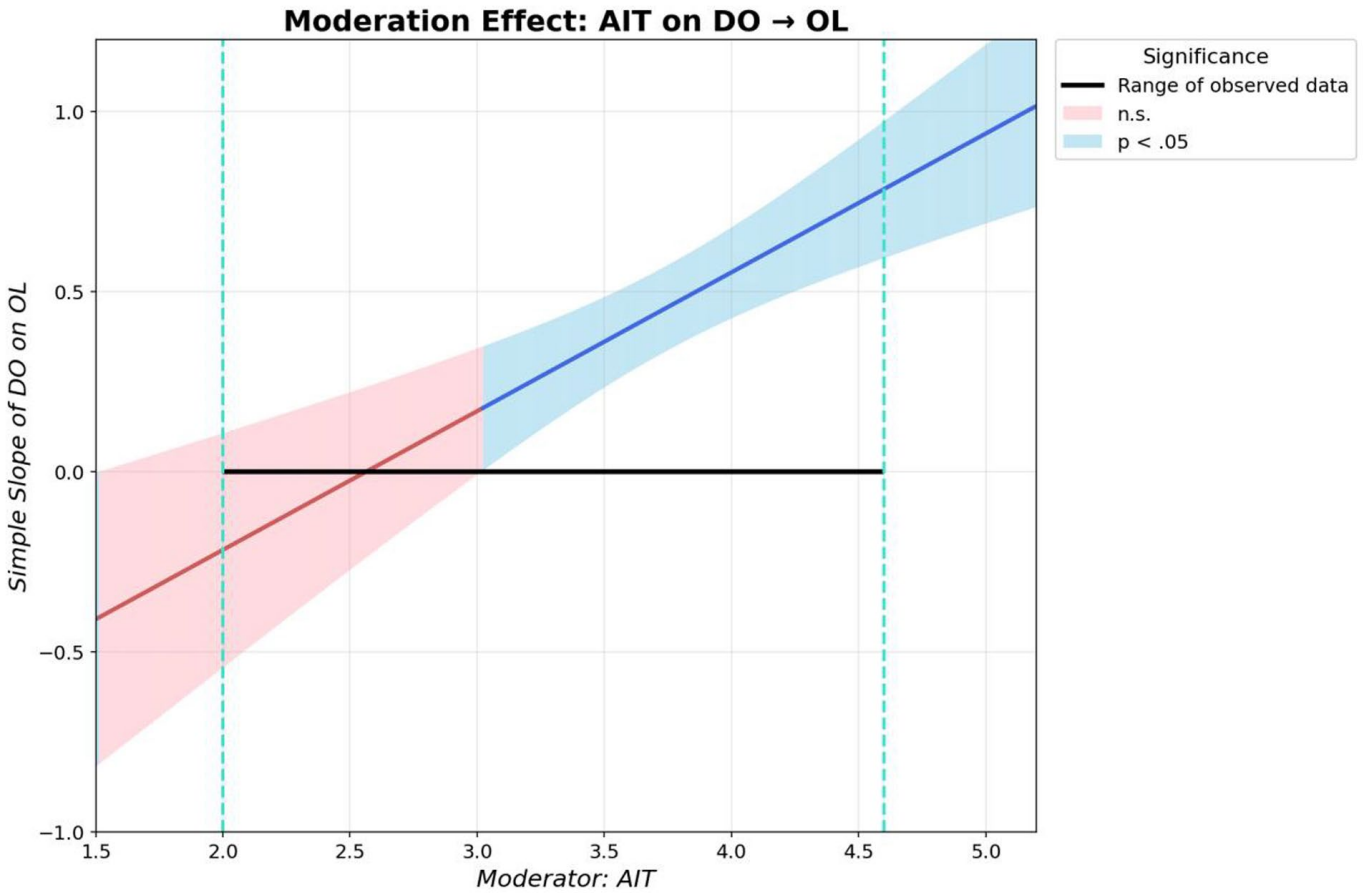
Johnson–Neyman plot of the conditional effect of digital orientation on organizational learning.

To further probe the form of the interactions, we applied the Johnson–Neyman technique to identify the regions of AI trust within which the conditional effect of digital orientation reached statistical significance. Figure 4 plots the simple slope of digital orientation on Organizational learning across the observed range of AI trust, together with the corresponding 95% confidence band. A single Johnson–Neyman critical value emerged at AIT = 3.03. Below this threshold the conditional effect was non-significant; above it, the effect became significant and positive, strengthening monotonically as AI trust increased.

**Figure 5:**
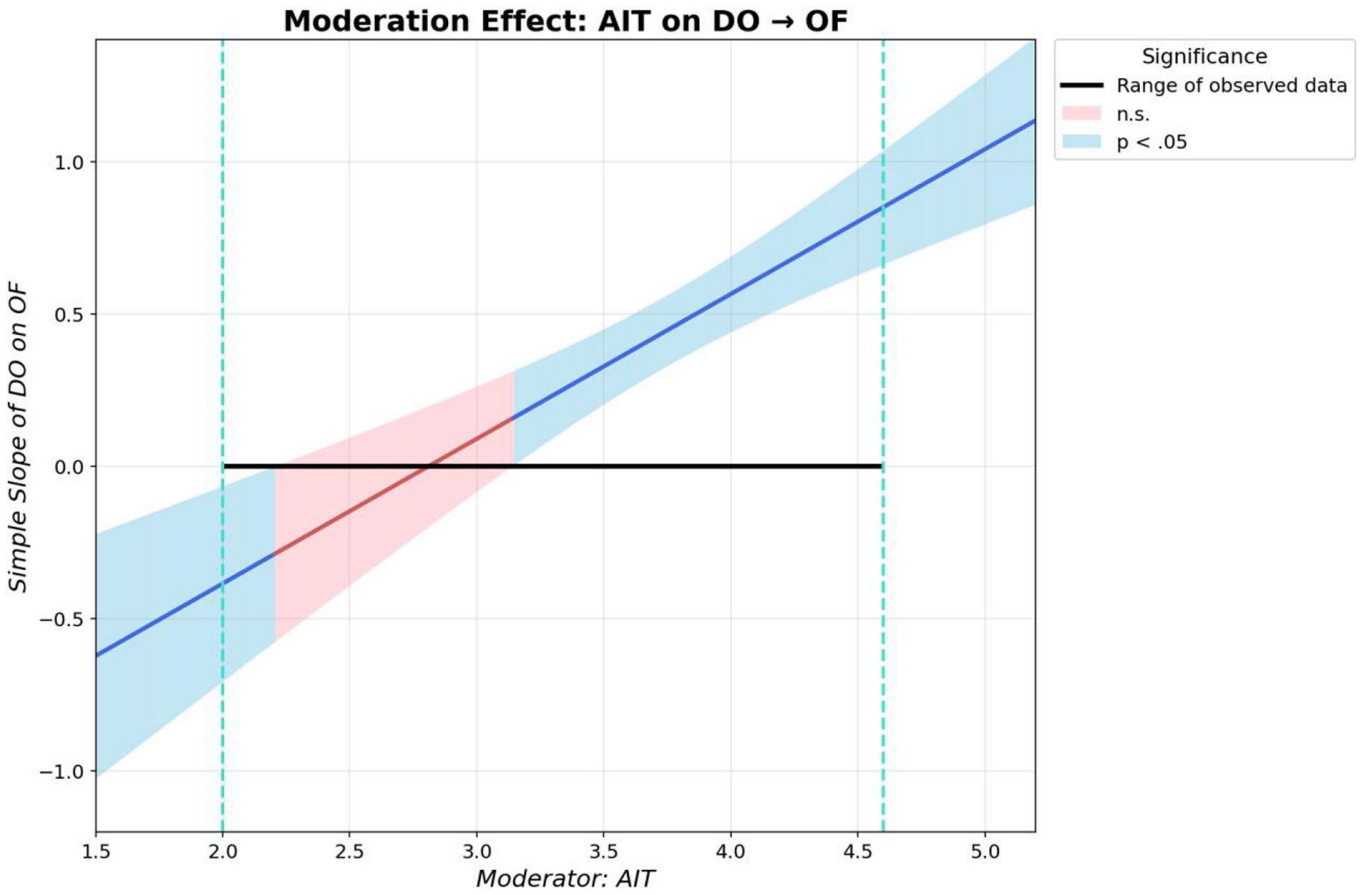
Johnson–Neyman plot of the conditional effect of digital orientation on organizational forgetting.

Figure 5 presents the analogous plot for the path from digital orientation to Organizational forgetting. Two Johnson-Neyman critical values emerged, at AIT = 2.17 and AIT = 3.18. Below 2.17 the conditional effect was significant and negative; between 2.17 and 3.18 it was non-significant; above 3.18 it became significant and positive. The pattern suggests that AI trust must accumulate past a certain threshold before digital orientation begins to prompt firms to discard outdated routines. The significant negative effect below the lower threshold (AIT = 2.17) warrants a cautious reading rather than a straightforward threshold interpretation. Substantively, it is consistent with defensive entrenchment: where trust in AI is very low, a strong digital push may lead members to treat established routines as a hedge against systems they do not trust, retaining or even reinforcing legacy practices rather than relinquishing them, which echoes the withdrawal and protective behaviors observed under low-trust conditions (Vuori et al., 2026). Methodologically, this region lies in the left tail of the trust distribution and rests on relatively few cases, so the negative estimate may also reflect imprecision or a suppression effect within the interaction structure. We therefore do not over-interpret it and read it primarily as evidence that the conversion of digital orientation into forgetting requires a minimum level of trust.

#### 4.3.3 The moderated mediation effect of AI trust

Building on the moderation findings, we tested the moderated mediation hypotheses by examining the conditional indirect effects of digital orientation on AI capability through Organizational learning and Organizational forgetting at low (−1 SD), mean and high (+1 SD) levels of AI trust, with 95% bias-corrected confidence intervals constructed from 5,000 bootstrap resamples (Hayes, 2018). Table 7 reports the results.

**Table 7:** Conditional indirect effects of digital orientation on AI capability at different levels of AI trust.

Mediator path	AI trust level	Indirect effect	S.E.	Bootstrap 95% CI
DO → OL → AIC	Low (−1 SD)	0.040	0.025	[−0.009, 0.092]
	Mean	0.117	0.031	[0.061, 0.181]
	High (+1 SD)	0.156	0.040	[0.082, 0.234]
DO → OF → AIC	Low (−1 SD)	0.018	0.029	[−0.052, 0.069]
	Mean	0.112	0.032	[0.054, 0.179]
	High (+1 SD)	0.158	0.043	[0.081, 0.248]

Bootstrap with 5,000 resamples. BC = bias-corrected.

For the indirect path through Organizational learning, the conditional effect was non-significant at low levels of AI trust (*β* = 0.040, Bootstrap 95% CI = [−0.009, 0.092]), became significant at the mean level (*β* = 0.117, Bootstrap 95% CI = [0.061, 0.181]), and strengthened further at high levels (*β* = 0.156, Bootstrap 95% CI = [0.082, 0.234]). The pattern was the same for the indirect path through Organizational forgetting: non-significant at low AI trust (*β* = 0.018, Bootstrap 95% CI = [−0.052, 0.069]), significant at the mean (*β* = 0.112, Bootstrap 95% CI = [0.054, 0.179]), and stronger at high levels (*β* = 0.158, Bootstrap 95% CI = [0.081, 0.248]). The monotonic strengthening of both indirect paths as AI trust rose confirms that AI trust positively moderates the indirect effects of digital orientation on AI capability through both mediators, providing support for H6 and H7.

### 4.4 Configurational analysis

Although the structural equation analysis quantifies the average net effect of each predictor on AI capability, it cannot identify which combinations of antecedent conditions are jointly sufficient for high AI capability. Drawing on Fiss (2007, 2011) and Misangyi et al. (2017), we complement the SEM with a fuzzy-set qualitative comparative analysis (fsQCA) that captures equifinality and conjunctural causation among the antecedents. The four focal conditions retained from the structural model, namely digital orientation (DO), Organizational learning (OL), Organizational forgetting (OF) and AI trust (AIT), were augmented with three firm-level contextual conditions (firm age, firm size and ownership type), and AI capability (AIC) served as the outcome. The analysis was implemented in fsQCA 3.0 (Ragin, 2008).

#### 4.4.1 Data calibration and necessary condition analysis

For the four continuous focal conditions and the outcome, we applied the direct calibration method, using the 95th, 50th and 5th percentiles of each variable’s distribution as the thresholds for full membership, the cross-over point and full non-membership, respectively, (Ragin, 2008). Because the focal conditions are perceptual constructs measured on Likert-type scales, no external substantive anchors are available; in this situation, percentile-based anchors derived from the sample distribution are a standard and widely used calibration approach in QCA research (Ragin, 2008), and we adopt the 95th/50th/5th convention accordingly. The three categorical control variables were calibrated using an indirect four-value scheme (1 → 0; 2 → 0.33; 3 → 0.67; 4 → 1) consistent with their original ordinal scaling. Calibrated values that exactly equaled 0.5 were adjusted upward by 0.001 to retain the corresponding cases in subsequent truth-table analyses (Fiss, 2011). Table 8 reports the calibration anchors.

**Table 8:** Calibration of conditions and outcome.

Type	Variable	Full membership	Cross-over	Full non-membership
Conditions	Digital orientation	4.20	3.60	3.00
	Organizational learning	4.80	3.80	3.00
	Organizational forgetting	4.20	3.20	2.80
	AI trust	4.40	3.80	3.00
	Firm age	Firm age is assigned 0 for 10 years and below, 0.33 for 11–15 years, 0.67 for 16–20 years, and 1 for 21 years and above.		
	Firm size	Firm size is assigned 0 for code 1, 0.33 for code 2, 0.67 for code 3, and 1 for code 4.		
	Ownership type	Ownership type is assigned 0 for code 1, 0.33 for code 2, 0.67 for code 3, and 1 for code 4.		
Outcome	AI capability	4.79	4.00	3.00

We then examined each antecedent for individual necessity. By convention, a condition is regarded as necessary when its consistency exceeds 0.90 (Schneider and Wagemann, 2012). As Table 9 shows, none of the seven conditions, whether in their presence or in their absence, reached this 0.90 threshold. No single condition therefore qualifies as necessary, and high AI capability must be understood as the joint outcome of multiple antecedents acting in combination, which motivates the configurational sufficiency analysis below.

**Table 9:** Necessary condition analysis for high AI capability.

Condition	Consistency	Coverage
Digital orientation	0.763	0.692
~Digital orientation	0.541	0.457
Organizational learning	0.770	0.732
~Organizational learning	0.622	0.504
Organizational forgetting	0.748	0.731
~Organizational forgetting	0.592	0.469
AI trust	0.696	0.666
~AI trust	0.604	0.486
Firm age	0.436	0.575
~Firm age	0.750	0.490
Firm size	0.614	0.583
~Firm size	0.643	0.521
Ownership type	0.603	0.668
~Ownership type	0.786	0.567

“~” denotes the absence of the condition.

#### 4.4.2 Configurational analysis of high AI capability

We performed the truth-table analysis with the raw consistency threshold set at 0.80, the PRI consistency threshold at 0.70 and the case-frequency threshold at 2 (Greckhamer et al., 2018). Following Ragin (2008) and Fiss (2011), the intermediate solution is reported as the primary configurational result, and the parsimonious solution is used to identify core conditions: a condition appearing in both solutions is treated as a core condition, while a condition appearing only in the intermediate solution is treated as a peripheral condition. In Table 10, “⚫” denotes the presence of a core condition, “•” the presence of a peripheral condition, “⊗” the absence of a core condition, and “⊗” the absence of a peripheral condition; a blank cell indicates that the condition is irrelevant in the corresponding pathway.

**Table 10:** Configurations for high AI capability.

Antecedent condition	S1	S2	S3	S4	S5
Digital orientation	⚫	⚫	⚫	⚫	⚫
Organizational learning	•	•	•		⚫
Organizational forgetting	⚫	⚫	⚫	⚫	•
AI trust	⚫	⚫	⚫	⚫	
Firm age		⊗		⊗	⚫
Firm size			•	•	•
Ownership type	⊗			⊗	⊗
Raw coverage	0.382	0.352	0.309	0.227	0.154
Unique coverage	0.038	0.034	0.005	0.018	0.015
Consistency	0.926	0.933	0.951	0.929	0.916
Overall coverage	0.489				
Overall consistency	0.915				

The analysis revealed five sufficient paths (S1–S5) to achieve high AI capability, with path consistencies all above 0.91 and an overall solution consistency of 0.915. All path consistencies were above 0.91. The overall solution consistency was 0.915. The overall solution coverage was 0.489. This means that the five pathways together explain about 49% of the high-AIC cases.

1. Trust–Forgetting Synergy Configuration. Paths S1 through S4 share three core conditions: digital orientation, Organizational forgetting, and AI trust. The internal logic is consistent across the four paths. A strong digital strategy provides direction; Organizational forgetting clears away entrenched knowledge structures and path dependencies; and AI trust lowers the psychological resistance to adopting AI in routine work. Together these elements produce what can be described as a trust–forgetting configuration. This label denotes the joint presence of these conditions, not a temporal sequence: fsQCA establishes which conditions are jointly sufficient for the outcome and which co-occur, but it does not establish the order in which they unfold, and we make no claim about process timing here. Organizational learning enters as a peripheral condition in S1, S2, and S3, where moderate learning appears to refill the knowledge voids opened by forgetting. In S4 it drops out entirely, suggesting that firms with limited prior knowledge stocks can sustain AI capability formation through the forgetting–trust mechanism alone. The four paths nevertheless diverge in scope. S1, with the highest raw coverage in the table at 0.382, applies across ownership types. S2 is concentrated in younger firms, S3 in medium- and large-sized firms, and S4 in younger firms of a certain scale that are not ownership-specific. This pattern reinforces the structural-model finding that Organizational forgetting acts as a substantive mediator and that AI trust positively moderates the indirect paths.
2. Mature-Firm Learning-Driven Configuration. Path S5 follows a different route. Its core conditions are digital orientation, Organizational learning and firm age; Organizational forgetting and firm size enter as peripheral conditions; AI trust is irrelevant. Mature firms in this configuration build AI capability through gradual learning-driven assimilation: long-accumulated learning routines and codified knowledge-management systems do most of the work, and the disruptive forgetting that drives the first pattern is largely absent. The peripheral role of Organizational forgetting, combined with the irrelevance of AI trust, suggests two things at once. In older firms, AI trust may already be embedded in Organizational culture and therefore discriminate weakly across cases. Learning capacity, by contrast, takes over as the dominant transmission mechanism. This configuration is consistent with the partial mediating role of Organizational learning identified in the structural model, and adds the further observation that firm age qualitatively shapes which route to AI capability a firm follows.

Three conditions emerge as especially consequential across the five sufficient pathways: digital orientation, Organizational forgetting, and AI trust. The first is ubiquitous. Digital orientation appears as a core condition in all five pathways, so that it appears consistently across all identified pathways to high AI capability. Organizational forgetting comes next in prominence, entering as a core condition in four of the five pathways, while Organizational learning enters as core in only one. The asymmetry is interpretively important: the capacity to discard obsolete routines is more frequently the binding requirement than the capacity to acquire new knowledge, a finding the SEM net effects cannot deliver on their own. AI trust, by contrast, shows clear context dependence. It is core in the Trust–Forgetting Synergy pattern but irrelevant in the Mature-Firm Learning-Driven pattern, implying that its activating role is contingent on firm maturity rather than universal. A robustness check raising the raw consistency threshold from 0.80 to 0.85 left the configurational solutions unchanged in composition and interpretation, indicating that these findings are not artefacts of threshold choice.

## 5 Discussion

Our results extend recent work that treats digital orientation as an antecedent of digital capability, innovation performance and organizational resilience (Ranjan, 2024; Zhang et al., 2025; Shen et al., 2024). What we add is more specific. Digital orientation is also associated with AI capability, and this association operates not as a direct strategic effect but through the knowledge processes by which firms acquire new digital knowledge and let go of routines that no longer fit AI-enabled work.

The mediation analysis supports treating AI capability development as a learning-intensive process rather than a technological investment decision alone (Pumplun et al., 2021; Wang Y. et al., 2025). But learning alone is incomplete. Following the organizational unlearning literature (Klammer et al., 2025; Maccioni and Ghiringhelli, 2025; Sharma and Lenka, 2025), we treat forgetting as a substantive complement to learning rather than a residual process in AI capability construction.

AI trust adds a third dimension. Prior research has identified trust as a central condition shaping organizational responses to AI (Glikson and Woolley, 2020; Gillespie and Lockey, 2025; Heimberger et al., 2026). Our findings sharpen this view by showing that AI trust strengthens the association between digital orientation and both organizational learning and organizational forgetting: firms are more likely to convert digital strategic intent into AI capability when their members perceive AI systems as reliable and useful. The fsQCA results add a configurational layer to the same picture. High AI capability can be reached through more than one route. One combines digital orientation, organizational forgetting and AI trust; another, more typical of mature firms, rests on digital orientation and organizational learning in a gradual, learning-based configuration. AI capability formation is therefore knowledge-dependent and context-contingent in ways the linear model alone does not capture. This dual dependence is bracketed by two recent strands of work: at the organizational level, evidence that AI readiness raises business-process efficiency mainly through an AI-driven transformation process rather than directly (Salam et al., 2026a) echoes our claim that readiness matters chiefly when carried through intervening processes, while at the individual level, acceptance research shows that willingness to rely on AI hinges on usefulness expectancy and is tempered by privacy concerns (Salam et al., 2026b), complementing our treatment of trust as the condition that lets digital orientation be enacted.

### 5.1 Theoretical implications

Three theoretical implications follow.

The first speaks to the digital orientation literature. Prior research has linked digital orientation to innovation performance, digital capability and Organizational resilience (Ranjan, 2024; Zhang et al., 2025; Shen et al., 2024), but the mechanisms running from digital orientation to AI capability specifically have received less attention. Our findings recast digital orientation as more than a strategic posture toward digital technologies. It functions as a knowledge-directing force that aligns search, routine reassessment and resource allocation with AI capability formation.

The study also contributes to the Organizational learning and unlearning literature by demonstrating that learning and forgetting operate as complementary mechanisms. Recent work has emphasized the role of Organizational learning in digital transformation and AI capability development (Pumplun et al., 2021), while a parallel literature has stressed that digital transformation also requires firms to abandon obsolete routines and path-dependent practices (Klammer et al., 2025; Maccioni and Ghiringhelli, 2025). Our results bring these two streams together by showing that Organizational learning and Organizational forgetting both transmit the effect of digital orientation to AI capability. AI capability is therefore built not only by accumulating new knowledge but also by selectively removing outdated knowledge that constrains AI-enabled renewal.

A third implication concerns AI capability research itself. The literature has long recognized AI capability as a multidimensional Organizational capability dependent on data, technology, human skills and managerial resources (Mikalef and Gupta, 2021). Our findings indicate that such capability does not emerge from resources alone. It also depends on whether Organizational members trust AI systems sufficiently to learn from them, work with them and reorganize routines around them. This evidence supports recent claims that trust, transparency and perceived reliability are central to organizational readiness for AI adoption (Gillespie and Lockey, 2025; Heimberger et al., 2026). AI trust is not only an attitude toward AI. It is also a condition that helps digital orientation turn into AI capability.

### 5.2 Practical implications

The findings also yield three practical implications for managers.

The first is that digital orientation must be translated into Organizational action. A firm may declare a strong commitment to digital transformation, but such commitment will remain largely symbolic if it does not change how employees learn, experiment and solve problems. Managers should therefore connect digital strategy with concrete mechanisms for AI experimentation, cross-functional knowledge sharing and digital capability development. This recommendation is consistent with recent evidence that digital orientation produces stronger Organizational outcomes when supported by capability-building processes (Zhang et al., 2025; Egala et al., 2024).

The second concerns how Organizational learning and Organizational forgetting should be managed together. Learning routines extend the organizational outward, asking employees to bring in new digital knowledge, interact with external sources, and experiment with AI applications. Forgetting works in the opposite direction. It asks the organizational to identify obsolete processes, question established routines, and remove practices that no longer fit AI-enabled work. Both are required because digital transformation faces entrenched routines and path dependencies as major obstacles (Maccioni and Ghiringhelli, 2025; Sharma and Lenka, 2025). Firms that learn without forgetting layer new technology onto old routines; firms that forget without learning lose direction. AI capability building therefore depends on both processes running in parallel.

The third concerns AI trust which should be treated as a strategic resource rather than a residual condition. Digital orientation becomes more effective when Organizational members trust AI systems. Firms should therefore work to improve the reliability, transparency and explainability of AI applications, provide employees with successful experiences of AI use, and establish governance mechanisms that reduce uncertainty around AI-generated outputs. This recommendation aligns with recent evidence that trust remains a central challenge in AI adoption and use (Gillespie and Lockey, 2025). Trust in AI should not be left to emerge spontaneously; it needs to be deliberately cultivated if firms expect employees to learn from AI, collaborate with AI systems and redesign routines around AI-enabled work.

Finally, these implications should be applied with attention to firm context, because the configurational results show that firms reach AI capability by different routes depending mainly on their age. Younger firms appear more often on the trust-and-forgetting route (paths S2 and S4), where the binding tasks are building members’ trust in AI and deliberately retiring routines that no longer fit AI-enabled work; for these firms, managerial effort is best directed at trust-building and at the active disposal of legacy practices. Mature firms, by contrast, appear on the learning-driven route (path S5), where long-accumulated learning routines and codified knowledge-management systems carry most of the load and disruptive unlearning is largely absent; here the priority is to channel AI initiatives through existing learning and assimilation mechanisms. Managers should therefore read the recommendations above through the route their firm is most likely to travel, given its maturity, rather than as a single uniform prescription. Whether these routes also differ systematically across industries is a question our data cannot settle, because the sample was not designed to compare sectors; we flag sector-stratified analysis as a promising direction for future research.

### 5.3 Limitations and future research

Our analysis has several limitations that suggest avenues for future research.

First, the data were drawn from Chinese enterprises, a context well-suited for studying AI capability formation in a rapidly digitalizing economy. Whether the mechanisms uncovered here generalize to other institutional and cultural contexts remains an open question for subsequent research.

Second, our analyses rely on cross-sectional survey data. Although the measurement model, common method tests and robustness checks provide support for the validity of the findings, longitudinal, archival or multi-source data could capture the dynamic process through which digital orientation shapes AI capability over time. We wish to state the implications of this design plainly. A single-source, cross-sectional design limits causal inference in three respects. First, although Harman’s single-factor test and the confirmatory factor comparison are reassuring, they are only partial safeguards and cannot fully rule out common method bias. Second, the design cannot establish temporal order, so reverse causality is possible: firms that have already developed stronger AI capability may, in turn, reinforce their digital orientation and their learning and forgetting routines. Third, unobserved factors, such as leadership quality or competitive pressure, could jointly drive the predictor and the outcome, raising the possibility of endogeneity. For these reasons we have framed our findings in associational rather than strictly causal terms. The most valuable next step is longitudinal and multi-wave research, ideally combined with archival or multi-source data, which would allow the temporal ordering implied by our theory to be tested directly and would trace how digital orientation, learning, forgetting and trust co-evolve into AI capability over time.

Third, AI capability has been treated here as an integrated Organizational capability. Future research could disaggregate this construct into its component dimensions, including data capability, algorithmic capability, managerial AI capability, human AI expertise and AI governance capability, and examine whether Organizational learning, Organizational forgetting and AI trust influence different dimensions of AI capability in distinct ways.

## 6 Conclusion

This study has examined how digital orientation contributes to the development of AI capability. Drawing on the attention-based view, we conceptualized digital orientation as a firm-level attentional configuration that channels managerial focus toward digital and AI-related issues. Strategic attention, however, becomes consequential only when it is carried into Organizational practice through knowledge-transformation processes. On this foundation, we examined the mediating roles of Organizational learning and Organizational forgetting, together with the boundary role of AI trust.

Drawing on survey data from 306 middle and senior managers in Chinese enterprises, our empirical analysis provides support for the proposed model. Digital orientation is positively related to AI capability, and both Organizational learning and Organizational forgetting transmit this relationship. Firms therefore build AI capability not only by acquiring new digital knowledge but also by releasing obsolete routines and assumptions. AI trust is associated with a stronger relationship between digital orientation and both learning and forgetting, and thus with stronger indirect pathways linking digital orientation to AI capability. The fsQCA results complement these findings by demonstrating that high AI capability can arise through multiple configurations, most notably a trust-and-forgetting synergy route and a mature-firm learning-driven route.

The evidence points to a layered picture of AI capability formation. It is neither a purely technological process nor a direct outcome of digital strategic intent, but emerges from the interaction among strategic attention, knowledge renewal and trust in AI systems. Firms seeking to build AI capability should therefore look past technology adoption itself and attend to the Organizational conditions under which digital orientation is converted into learning, forgetting and capability development. Future work could extend the framework with longitudinal data that captures how these conditions evolve, cross-country samples that test the boundaries of the present results, and finer-grained measures of the dimensions of AI capability identified here.

## Data Availability

The original contributions presented in the study are included in the article/supplementary material, further inquiries can be directed to the corresponding author/s.
